# Dendritic Cells Induce a Subpopulation of IL-12Rβ2-Expressing Treg that Specifically Consumes IL-12 to Control Th1 Responses

**DOI:** 10.1371/journal.pone.0146412

**Published:** 2016-01-08

**Authors:** Uri Sela, Chae Gyu Park, Andrew Park, Peter Olds, Shu Wang, Ralph M. Steinman, Vincent A. Fischetti

**Affiliations:** 1 Laboratory of Cellular Physiology and Immunology, Chris Browne Center for Immunology and Immune Disease, The Rockefeller University, New York, NY, 10065, United States of America; 2 Laboratory of Bacterial Pathogenesis and Immunology, The Rockefeller University, New York, NY, 10065, United States of America; 3 Laboratory of Immunology, Brain Korea 21 PLUS Project for Medical Science, Severance Biomedical Science Institute, Yonsei University College of Medicine, Seoul, 03722, Republic of Korea; Jackson Laboratory, UNITED STATES

## Abstract

Cytokines secreted from dendritic cells (DCs) play an important role in the regulation of T helper (Th) cell differentiation and activation into effector cells. Therefore, controlling cytokine secretion from DCs may potentially regulate Th differentiation/activation. DCs also induce *de-novo* generation of regulatory T cells (Treg) that modulate the immune response. In the current study we used the mixed leukocyte reaction (MLR) to investigate the effect of allospecific Treg on IL-12, TNFα and IL-6 secretion by DCs. Treg cells were found to markedly down-regulate IL-12 secretion from DCs following stimulation with TLR7/8 agonist. This down-regulation of IL-12 was neither due to a direct suppression of its production by the DCs nor a result of marked DC death. We found that IL-12 was rather actively consumed by Treg cells. IL-12 consumption was mediated by a subpopulation of IL-12Rβ2-expressing Treg cells and was dependent on MHC class-II expressed on dendritic cells. Furthermore, IL-12 consumption by Tregs increased their suppressive effect on T cell proliferation and Th1 activation. These results provide a new pathway of Th1 response regulation where IL-12 secreted by DCs is consumed by a sub-population of IL-12Rβ2-expressing Treg cells. Consumption of IL-12 by Tregs not only reduces the availability of IL-12 to Th effector cells but also enhances the Treg immunosuppressive effect. This DC-induced IL-12Rβ2-expressing Treg subpopulation may have a therapeutic advantage in suppressing Th1 mediated autoimmunity.

## Introduction

T cell differentiation into effector Th cells in response to an antigen is stimulated by DCs together with cytokines. For example, for Th1 cell differentiation, DCs provide the IL-12 required by the Th cells [1–5]. The various types of Th cells provide resistance to different types of infection but also mediate unwanted reactions such as autoimmunity, allergy and transplant rejection [6–8]. Therefore, regulating cytokine secretion from DCs would be important in modulating Th cell activation and differentiation and subsequently to achieve remission in some of these pathological conditions.

Naïve CD4+ cells can also be induced to become regulatory T cells (iTreg) upon stimulation with an antigen presented by DCs in the presence of TGFβ [9, 10]. The combined presence of TGFβ and all-trans-retinoic-acid (ATRA) enhances the induction of alloreactive Treg from the polyclonal CD4+ T cells [11]. These *de-novo*-generated iTreg exert substantial specificity in suppressing allogeneic DC both in culture and in a mouse model of graft vs. host disease [11]. Treg cells can inhibit T cell function by several mechanisms [12, 13], some of which require direct Treg suppression of DCs. This suppression involves down-regulation of CD80 and CD86 expression on DCs [14, 15] and up-regulation of its indoleamine 2,3-dioxygenase (IDO) activity [16]. These effects of Treg on DCs are mediated by the co-inhibitory molecule CTLA-4, [16, 17] a major molecule with which Treg regulate antigen presenting cell (APC) function [18], and by a possible effect of IL-10 secreted from Treg [19]. However, the direct effect of Treg in general and more specifically of iTreg on cytokine secretion by DCs is less well established.

The MLR system enables both the induction of Treg from the polyclonal repertoire of CD4+ T cell population and the evaluation of their specificity to the inducing allogeneic DCs [11]. In the present study we used the MLR system [11] to investigate whether allospecific polyclonal iTreg cells can regulate DC secretion of IL-12, TNFα and IL-6, as well as its mechanism and effect on the Th response.

## Results and Discussion

### DC-Induced Treg Cells Specifically Down-Regulate IL-12 Secreted from DCs by Its Consumption

**Fig 1:**
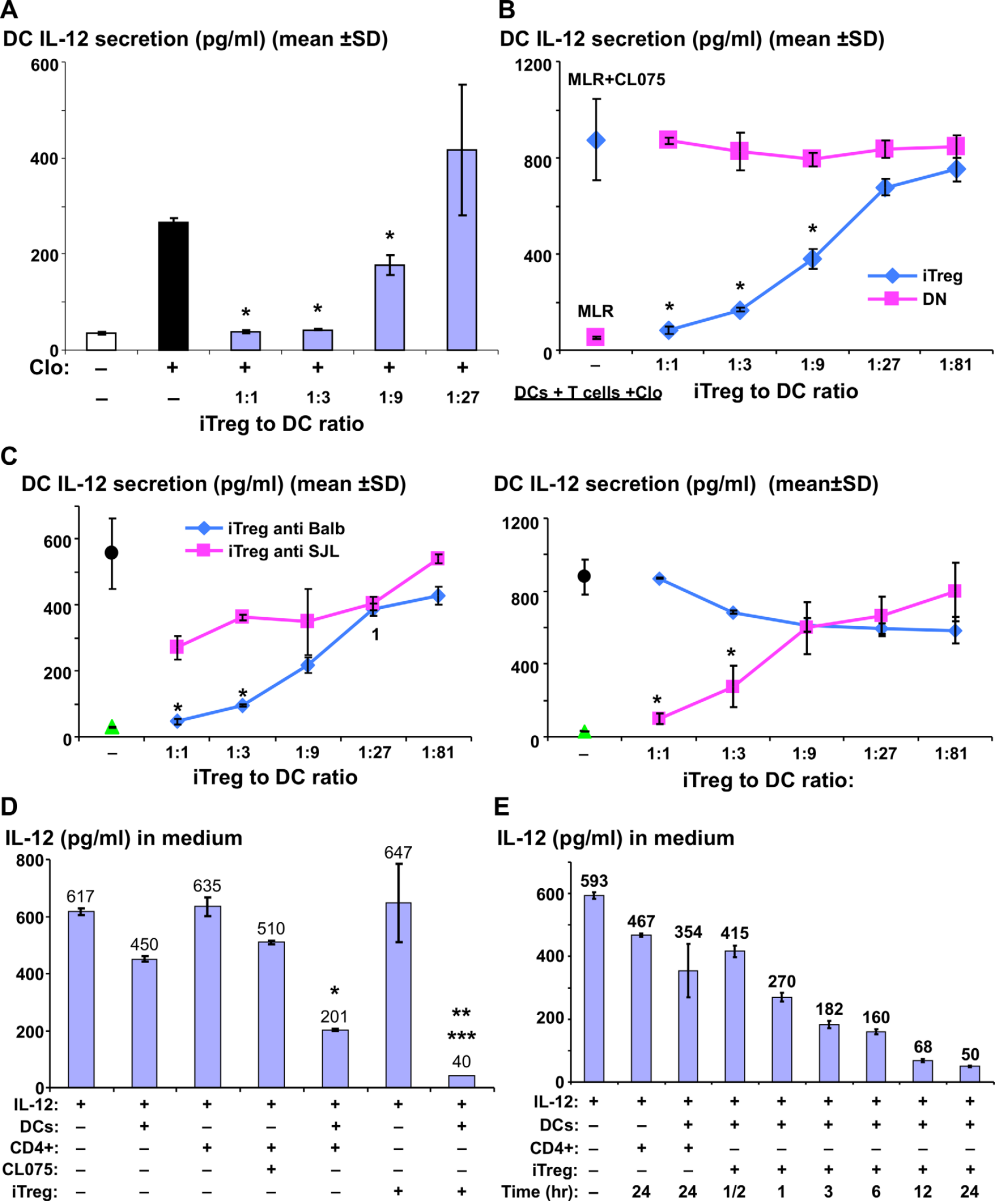
DC induced Treg specifically down-regulate IL-12 secreted from DC by its consumption. (A) Balb/c DCs were incubated (24hr) either alone or with various ratio of C57Bl/6 iTreg that were induced with Balb/c DCs (iTreg anti Balb). CL075 (1 μg/ml) was added to the culture and 24 hr later the level of secreted IL-12 was measured by ELISA. (B) Co-culture of Balb/c DCs and C57Bl/6 CD4+ T cells (MLR) was incubated alone or co-incubated with either anti-Balb/c iTreg or CD25^-^Foxp3^-^ (RFP^-^) (Double Negative, DN) cells for 24 hrs after which CL075 was added and IL-12 measured as in A. (C) Co-culture of CD4+ T cells and either Balb/c DCs (left panel) or SJL DCs (right panel) were incubated alone or co-incubated with B6 iTreg cells induced with either Balb/c DCs (iTreg anti Blab/c) or SJL DCs (iTreg anti SJL), after which CL075 was added and IL-12 was measured as in A. (D) IL-12p70 (800pg/ml) in culture medium was incubated alone or with either DCs, CD4+ T cells, or iTreg alone or with a co-culture of DCs and either CD4+ or anti Balb iTreg. Levels of IL-12 that remained in the medium were measured 48hrs later by ELISA. [*, ** = compared to medium with IL-12, *** = compared to co-culture of DCs and CD4+ in medium with IL-12]. (E) Time kinetics of IL-12 level in a medium added with either IL-12 alone or with DCs and anti- Balb iTreg. One representative experiment of three is shown. The consumption assay is one representative out of 6. Error bars denote mean ± SD. *, *** = P < 0.05, ** = P<0.001.

To determine if iTreg cells reduce cytokine secretion by DCs, we first used Balb/c DCs to induce allospecific iTreg from C57Bl/6 (B6) Foxp3^-^ CD4+ cells as described previously {[11], and S1 Fig}. The iTreg were then incubated with fresh Balb/c DCs for 24 hrs, after which TLR 7/8 ligand Thiazoloquinolone (CL075, 3M002) was added for an additional 24 hrs. CL075 induced IL-12 release from DCs, but this was completely blocked when iTreg were added (Fig 1A). In contrast, TNFα release was only mildly reduced by adding iTreg (up to 30%, S2 Fig).

To test whether suppression of IL-12 production also occurred when iTreg were suppressing an immune response, we added the iTreg to Balb/c DC along with fresh B6 CD4+ cells. The MLR between B6 T cells and Balb/c DC produces a powerful immune response but does not detectably result in IL-12 release unless CL075 was added (Fig 1B). The main source for IL-12 production and release into the medium was the DCs as expected (S3 Fig right and left). Addition of iTreg but not CD4+CD25-Foxp3- (DN) cells significantly down-regulated IL-12 secretion in a dose dependent manner (Fig 1B). However, production of two other cytokines, TNFα in response to CL075 and IL-6 in response to LPS, was only slightly reduced (~30%) following the addition of iTreg (S4 Fig right and left). Thus, based on these results, one functional effect of iTreg is to specifically suppress DC by reducing the level of secreted IL-12 in response to a TLR7/8 agonist.

To evaluate the specificity of the suppressive function of iTreg on IL-12 secretion from DCs, we generated iTreg cells by co-culturing B6 CD4^+^CD25^-^Foxp3^-^ cells (double negative) with either allo Balb/c DCs or SJL DCs in the presence of TGF-β and ATRA. The sorted CD4^+^CD25^+^Foxp3^+^ cells (double positive), termed “iTreg anti Balb/c” (when induced with Balb/c DCs) or “iTreg anti SJL” (when induced with SJL DCs), were shown previously to specifically suppress an MLR induced by either Balb/c or SJL DCs [11]. Here we found that simultaneously with suppression of T cell proliferation in the context of the MLR (data not shown), the iTreg also down-regulated CL075-induced IL-12 secretion from DCs specifically. These “iTreg anti Balb/c” cells down regulated CL075-induced IL-12 secretion from Balb/c DCs more effectively than “iTreg anti SJL”, and vice versa for IL-12 secretion from SJL DCs. (Fig 1C left and right, respectively). Specificity was further evidenced in the finding that “iTreg anti Balb/c” significantly down-regulated the CL075-induced IL-12 secretion from Balb/c DCs, but did not affect IL-12 secretion from B6 DCs (Fig 1C, S5 Fig). On the other hand, when added to F1 (Balb/c X B6) DCs, “iTreg anti Balb/c” significantly down-regulated CL075-induced IL-12 secretion in the same manner as it affected Balb/c (Fig 1C, S5 Fig). We conclude that iTreg selected on allogeneic DC of one type, specifically suppress IL-12 from the same type of DCs, in the presence of alloreactive T cells.

To understand more about the mechanism for iTreg suppression of IL-12 release from DCs, we tested blocking antibodies to several components that are required for different aspects of iTreg formation and function [12, 15, 17]. The antibodies were added to co-cultures of Balb/c DC, anti Balb/c specific iTreg, and B6 MLR responsive T cells, along with CL075. Anti-MHC class II mAbs (N22) abrogated the iTreg effect on IL-12 production, whereas the control hamster Ig isotype did not (S6 Fig upper panel). In contrast, blockade of TGFβ (S6 Fig, right lower panel) and blockade of CTLA4 (S6 Fig, left lower panel) or LFA-1 (data not shown), did not restore IL-12 release into the culture medium. Thus, the capacity of iTreg to block IL-12 release from DC is dependent on MHC II.

To assess if the down-regulation of IL-12 reflects increased dendritic cell death, as recently reported in an in vivo model [20], we stained cultures of DCs and T cells incubated with or without iTreg for viability. As shown in S7A Fig, co-incubation with iTreg decreased the number of live DCs by 30%. This reduction may explain the modest decrease in TNFα and IL-6 that we observed (S4 Fig), but seems unlikely to explain the almost 10 fold reduction in IL-12 secreted by DCs (Fig 1B).

To determine if the reduction in IL-12 measured from DC culture was due to suppression of its synthesis, we looked directly at IL-12 transcription and intracellular IL-12. S7B and S7C Fig demonstrate minimal changes in levels of IL-12 p40 mRNA and protein in the DCs. Thus, to our surprise, the ability of iTreg to suppress IL-12 release is not likely due to the toxicity or reduced IL-12 synthesis by DCs per se.

Given the above findings we searched for a new IL-12 control mechanism, whereby IL-12 would be consumed as a result of DC-iTreg interaction. We first added 800 pg/ml IL-12 to a co-culture of DCs with either naïve CD4+ cells or iTreg in the absence of CL075 stimulation. This is the concentration reliably secreted by DCs in our culture system stimulated by CL075 (Fig 1B). Addition of this amount of IL-12 to co-cultures of DCs and CD4+ T cells was followed by a 3-fold reduction in IL-12 levels (201 pg/ml and 617 pg/ml with or without a co-culture of DCs and CD4+ T cells, respectively). However, addition of IL-12 to a co-culture of DCs and iTreg cells resulted in a 12-fold reduction in IL-12 levels (50 pg/ml and 617 pg/ml with or without a co-culture of DCs and iTreg cells, respectively). This 12-fold reduction required the presence of both DCs and iTreg cells (Fig 1D), while each cell type alone resulted in either mild or no reduction in the level of IL-12 (450 pg/ml and 647 pg/ml, respectively). To further support the notion of IL-12 consumption by iTreg cells we wanted to evaluate the time course of removal of the added IL-12 from the media. The time required for IL-12 consumption was relatively short, as a two fold reduction was achieved within 0.5–3 hrs of co-incubation of DCs and iTreg cells in the IL-12 added media, and a maximum reduction of 10–12 fold after 12–24 hrs (Fig 1E). A rough estimate of IL-12 consumed per cell was 0.0005 pg IL-12 per cell in 12 hrs or ~6 molecules/min/cell [(800 pg/ml - 50pg/ml) x 0.2 ml /300,000 cells = 0.0005 pg/cell]. On the other hand, iTreg cells alone or co-incubated with DCs did not reduce TNFα when added to the medium (S8 Fig). The fact that the IL-12 level continues gradually and consistently to drop over time to almost complete removal (Fig 1E), rules out the possibility of saturation of IL-12 binding, in which we would expect a “plateau effect” after an initial steep non-gradual decline. However, the continued drop in IL-12 concentration support a possible mechanism of receptor uptake and recycle.

### IL-12 Consumption Is Mediated by Treg IL-12Rβ2 and Dependent on DC MHC-Class-II

**Fig 2:**
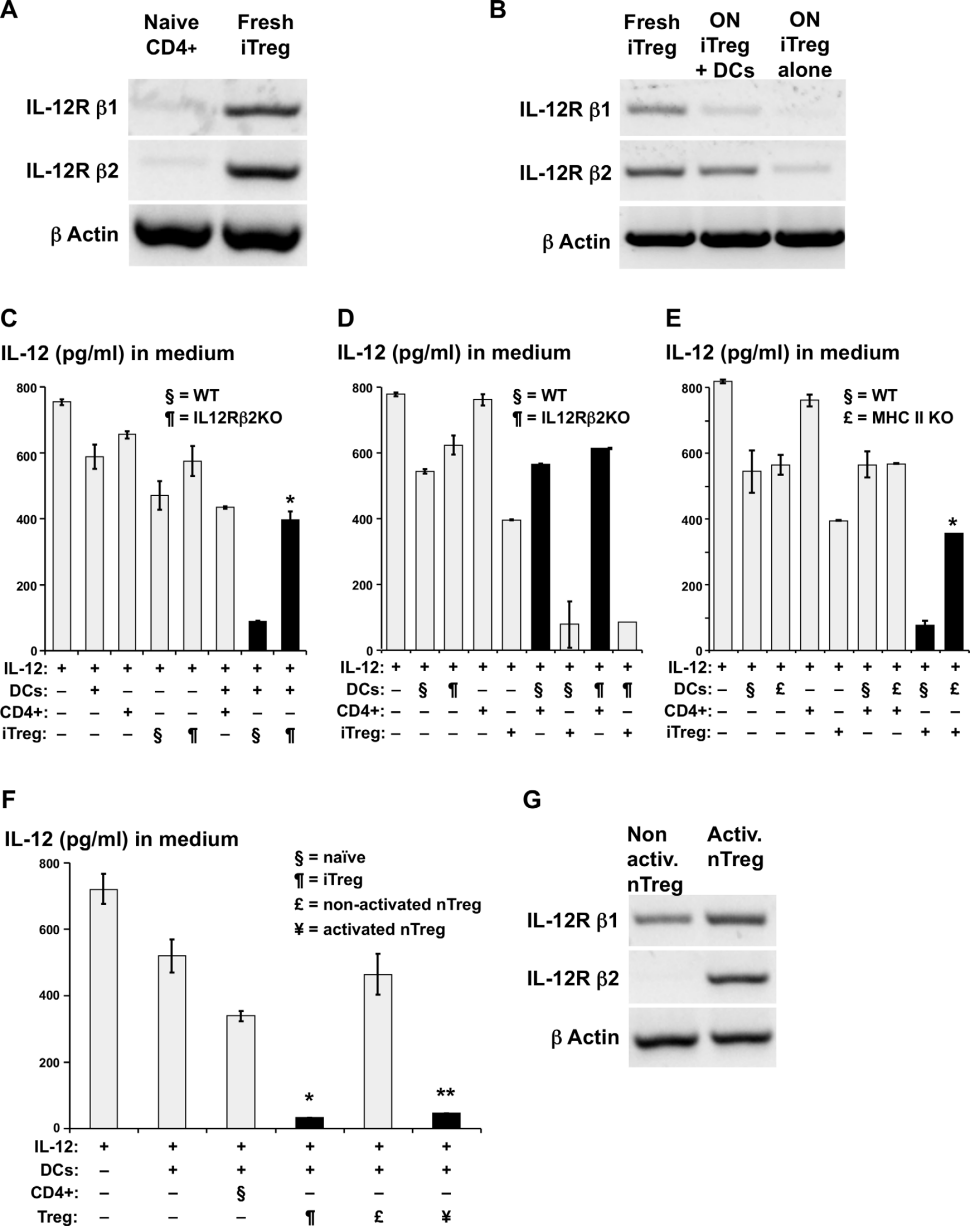
IL-12 consumption is mediated by Treg IL-12Rβ2 and dependent on DC MHC-Class-II. (A) RNA was isolated from either sorted naïve CD4+ T cells (CD4^+^CD25^-^Foxp3^-^) or fresh iTreg (CD4^+^CD25^+^Foxp3^+^). Synthesized cDNA of IL-12Rβ1, β2 and β Actin was amplified with PCR. The Agarose gel electrophoresis of the PCR products is shown. (B) As in A, but compared are PCR products from either fresh sorted iTreg, iTreg that were incubated overnight (ON) with Balb/c DCs or iTreg that were incubated overnight alone. (C) As in Fig 1D but a culture of DCs and either WT or IL-12Rβ2 KO iTreg (both induced with Balb/c DCs from CD4^+^CD25^-^Foxp3^-^ cells as described before) was added to the medium containing IL-12. (* = compared to co-culture WT iTreg and DCs in a medium of IL-12). (D) As in C but a culture of iTreg and either WT or IL-12Rβ2 KO Balb/c DCs was added to the medium containing IL-12. (E) As in C but a culture of iTreg and either WT or MHC class-II KO DCs was added to the medium containing IL-12. (* = compared to co-culture of iTreg and WT DCs in a medium of IL-12). (F) As in C but a culture of DCs and either fresh sorted non activated nTreg (CD4^+^CD25^+^Foxp3^+^) or nTreg that were sorted after activation in co-culture with allo-Balb/c DC were added to a medium with IL-12. (* = compared to medium with IL-12, ** = compared to co-culture of DCs and non-activated nTreg in medium with IL-12). (G) As in A, but non-activated to activated nTreg are compared. One representative experiment of three is shown. Error bars denote mean ± SD. *, ** = P < 0.05.

To test the possibility that IL-12 consumption was mediated by binding to IL-12R on the iTreg cells, we compared iTreg and naïve CD4+ cells for IL-12R β1 and β2 mRNA expression by RT-PCR. As shown in Fig 2A, iTreg cells sorted and analyzed immediately after their induction had higher levels of IL-12Rβ1 and even more IL-12Rβ2 compared to naïve CD4+ cells. To pinpoint the IL-12R expressing cells, we sorted iTreg that were co-cultured overnight either alone or with DCs, and compared their IL-12R expression to that of iTreg immediately after their induction. When iTreg were incubated alone overnight, expression of mRNA was greatly reduced for both IL-12R subunits, whereas iTreg cultured with DCs retained IL-12R mRNA (Fig 2B). To prove that IL-12Rβ2 was responsible for IL-12 consumption, we compared iTreg from IL-12Rβ2 KO mice (Foxp3^-^IRES^-^RFP (FIR) knock-in crossed with IL-12Rβ2 KO) to WT-iTreg. Indeed, the IL-12Rβ2 KO-iTreg were unable to consume IL-12 (Fig 2C) whereas DCs from IL-12Rβ2 KO mice incubated with WT iTreg were fully capable of inducing iTreg to consume IL-12 (Fig 2D), ruling out the possibility that IL-12 was consumed by DCs.

To verify whether IL-12 consumption was dependent on an interaction with MHC class-II, WT iTreg were incubated with DCs from MHC-class-II-KO mice. Lack of MHC-II on DCs abrogated the consumption of IL-12 achieved by iTreg incubated with WT-DCs (Fig 2E). Taken together, these results demonstrate that MHC-II expression on DCs and IL-12Rβ2 expression on iTreg are essential for the IL-12 consumption mechanism by iTreg.

We next tested if DCs can induce naturally occurring Treg (nTreg) to consume IL-12. To this end, we added IL-12 to a co-culture of Balb/c DCs with either freshly isolated CD4^+^CD25^+^Foxp3^+^nTreg, or nTreg that were sorted after activation and expansion with allogeneic DCs in an MLR system as described [11]. We found that during the MLR, DC-activated but not freshly isolated nTreg consumed IL-12 (Fig 2F), and much like DC-induced iTreg the nTreg also up-regulated IL-12Rβ2 (Fig 2G).

### IL-12Rβ2 Expressed on Authentic Treg Contribute to the Control of Th1 Response by Effector T Cells while Mediating IFNγ Secretion in a Stat4 Dependent Manner

**Fig 3:**
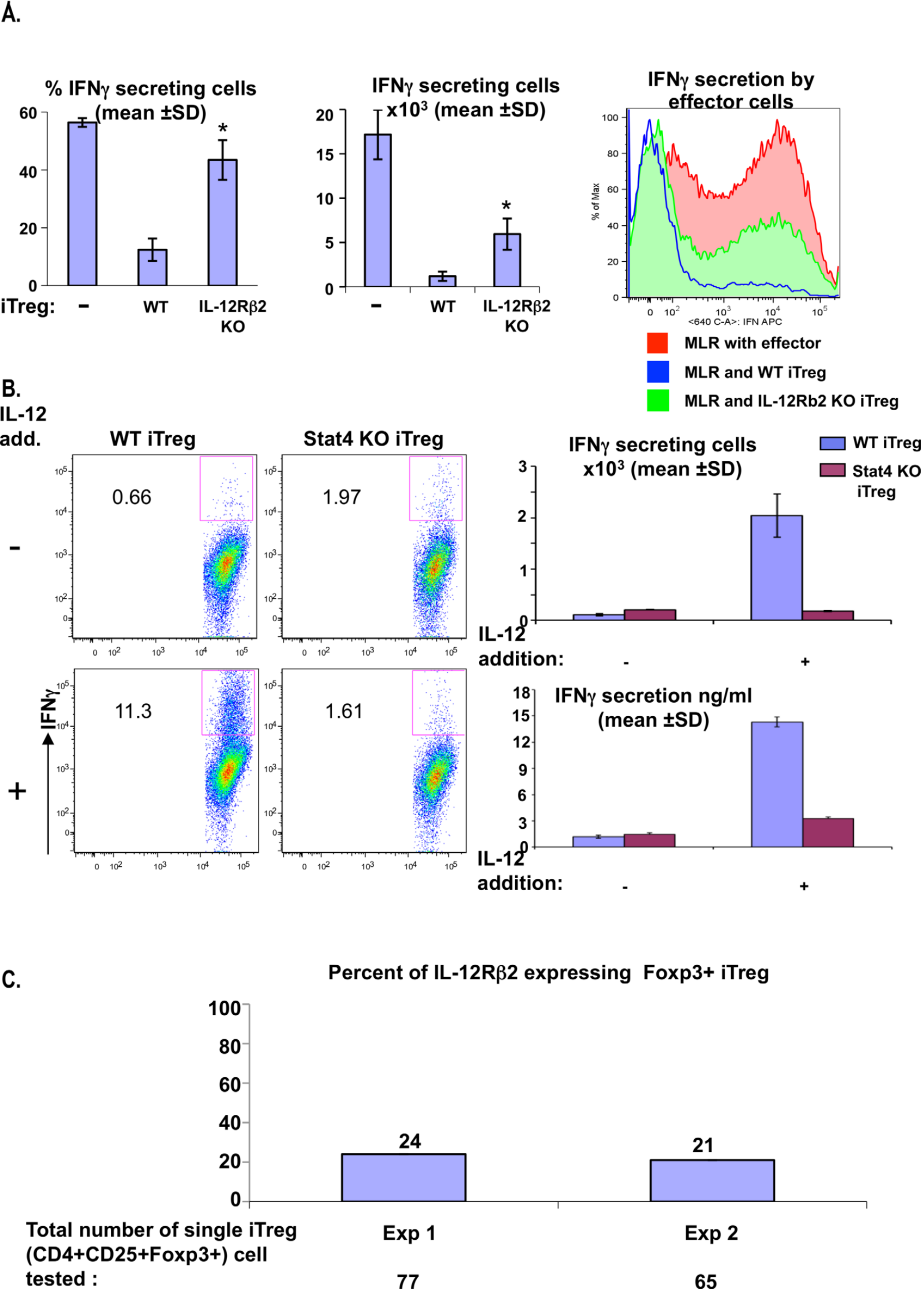
IL-12Rβ2 expressed on authentic Treg contribute to the control of Th1 response in a Stat4 dependent manner. (A) MLR culture of Balb/c DCs and sorted CFSE labeled effector CD4^+^CD25^+^Foxp3^-^cells (following naïve CD4^+^CD25^-^ pre-activation with Balb/c DCs for 3 days) was incubated with IL-12 (1000 pg/ml) alone or with either WT or IL-12Rβ2 KO iTreg cells for 24 hrs, and the culture was stained and analyzed for IFNγ secreting cells by FACS. Error bars denote mean ± SD. * = P < 0.05 (compared to WT) (B) Balb/c DCs were cultured with either WT or Stat4 KO sorted anti Balb/c iTreg. The culture was either stimulated (left lower panels) or not stimulated (left upper panels) with IL-12, and 24 hrs later stained and analyzed (left panel) for IFNγ expressing Treg cells (CD4+CD25+Foxp3+(RFP+) by FACS (absolute number in right upper panel), and for IFNγ secreted to the medium by ELISA (right lower panel). (C) WT CD4+CD25-Foxp3-(RFP-) were sorted, co-cultured with Balb/c DCs in the presence of TGFβ and ATRA. After 5 d CD4+CD25+Foxp3+(RFP+) cells were sorted and re-cultured with Balb/c DCs for re-stimulation. After 18 hr CD4+CD25+Foxp3+(RFP+) cells were re-sorted as a single cell per well. Each single cell was subjected to cDNA synthesis followed by nested PCR for the detection of Foxp3 and IL-12Rβ2 gene expression. The percent values for IL-12Rβ2 RNA expressing iTreg cells amongst the Foxp3 RNA expressing iTreg cells, respectively from 2 independent experiments, are indicated above each histogram.

To determine the importance of IL-12 consumption on the immune response, we added IL-12 (1000 pg/ml) to cultures of allo DCs (Balb/c) and CD4+ T cells (B6). In the absence of IL-12, iTreg suppressed the MLR in a dose dependent manner (S9A Fig). Since IL-12 was shown to increase T cell proliferation [21, 22], we expected a reduced suppression of pre-activated/effector T cell proliferation by iTreg in the presence of IL-12. However, the addition of IL-12 surprisingly enhanced the inhibitory effect of the iTreg (S9A Fig upper and lower panel). Further, this enhanced suppressive effect of iTreg in the presence of IL-12 was IL-12Rβ2 dependent. Indeed, WT iTreg suppressed T cell proliferation (S9B Fig) and IFNγ secretion (Fig 3A) from effector CD4+ cells several fold greater (2–3 and 3.5–5, respectively) than IL-12Rβ2 KO Treg. Moreover, the suppressive effect of WT iTreg (IL-12Rβ2 expressing) in the presence of IL-12 on the total number of IFNγ effector CD4+ secreting cells was 2-fold greater than the effect on the total number of proliferating cells [~10-fold (Fig 3A middle) versus ~5-fold (S9B Fig middle) reduction, respectively, compared to MLR with effector CD4+ cells]. These results support a direct effect of Treg on effector Th1 response and IFNγ secretion beyond and possibly independent of the suppression of effector CD4+ proliferation. Together, these results may suggest that IL-12, a cytokine with effector immuno-stimulatory properties, gains suppressive function in the presence of IL12Rβ2-expressing iTreg. Our results may also explain cases in which single-nucleotide polymorphisms (SNPs) in IL-12, IL-12Rβ2, and Stat-4 were shown to increase the risk for diseases such as primary biliary cirrhosis [23], in which Treg, and IL-12 are part of the pathogenesis. This may raise the possibility that the presence of the same SNPs on IL-12Rβ2 on iTreg abrogate their suppressive function.

IL-12 addition to a culture of nTreg cells was shown to induce IFNγ secretion through an unclear pathway [24, 25]. To verify that iTreg (induced with DCs in MLR) can secrete IFNγ, and to explore whether IFNγ secretion by iTreg is mediated via IL-12R expression, we studied the stimulatory effect of this receptor on cytokine secretion by iTreg. As was shown recently with nTreg [26], here we demonstrate that addition of IL-12 to a culture of DCs and allospecific iTreg, induced marked up-regulation in IFNγ secretion from WT iTreg but not from IL-12Rβ2 KO iTreg cells (S10 Fig). Stat4 and T-bet act independently and synergistically to drive expression of the signature inflammatory cytokine IFN-γ [27, 28]. While it was demonstrated that Tbet+ nTreg undergo abortive Th1 cell differentiation due to the impaired expression of IL-12 receptor β2 [29], here we demonstrate that the IFNγ secretion from iTreg was dependent on the transcription factor Stat-4 (Fig 3B) which resides downstream of the IL-12R signaling pathway.

At this point we wanted to verify that the IL-12Rβ2-expressing cells are authentic Tregs and not ex-Foxp3 effector T cells [30]. In the absence of a reliable antibody against IL-12Rβ2, a culture of WT iTreg cells incubated with DCs, were sorted for CD4^+^CD25^+^Foxp3^+^ (RFP^+^) single cells. By analyzing single cells for the concomitant expression of IL-12Rβ2 and Foxp3 mRNA, we could define a relatively abundant subpopulation (20–25%) of IL-12Rβ2 expressing Foxp3+ iTreg cells in WT mice (Fig 3C).

Recent studies have shown that Foxp3+ Treg cells can functionally differentiate to acquire the ability to specifically control Th1, Th2 or Th17 type inflammatory responses of certain T helper types by modifying their expression of related transcription factors [31–33]. For example, Treg that acquired T-bet expression have been shown to control Th1 responses, though by a yet an unknown mechanism [31]. Here we show that a subpopulation of iTreg cells acquire the expression of IL-12R thereby contributing to the regulation of Th1 responses. Interestingly, this subpopulation of Treg cells uses the same receptor as effector Th1 cells in order to suppress Th1 responses. Simultaneously, the effector Th1-suppressive iTreg secrete IFNγ (in a Stat4 dependent manner), a cytokine, that when secreted from Treg, was shown recently to be suppressive in the context of graft versus host disease (GVHD) and transplant rejection [26, 34]. More specifically, Koenecke et al [26] have shown that only IFNγ-expressing and not IFNγ-deficient Treg could suppress GVHD. Taken together, IFNγ produced by iTreg is dependent on the IL-12/IL-12Rβ2/Stat4 pathway, and may further add to the suppression achieved by the IL-12Rβb2.

In summary, we describe here a new pathway whereby DCs induce the expression of IL-12R on a subpopulation of Treg cells, in an MHC class II dependent manner. This novel mechanism contributes to the regulation of Th1 effector response by iTreg through the consumption of IL-12, which in this milieu becomes a cytokine with an immunosuppressive outcome. These DC-induced IL-12Rβ2-expressing Treg cells may have a significant therapeutic advantage in Th1 mediated autoimmunity.

## Material and Methods

### Mice

6–8-wk-old female Balb/c H-2^*d*^, C57BL/6 CD45.1 H-2^*b*^, and SJL H-2^*s*^ mice are from Taconic. MHC class-II, IL-12Rβ2 knock-out, IL-12b (p40)-IRES-eGFP knock-in mice are from Jackson laboratories. Foxp3^-^IRES^-^RFP (FIR) knock-in mice were a gift from R. Flavell (Yale University, New Haven, CT; [35] and were crossed with IL-12Rβ2 knock-out mice (Jackson lab) for studying IL-2Rβ2 knock-out CD4^+^Foxp3^+^(RFP^+^) cells. Stat-4 knock-out mice (Jackson lab) were crossed with Foxp3^-^GFP knock-in mice (Jackson lab) for studying Stat-4 KO CD4+Foxp3+(GFP+). Mice housing and husbandary was in Rockefeller University animal fascility, with regular diet and caging. The study was approaved by institutional animal care and use committee of the Rockefeller University, and we followed its guidelines. All experiment were done ex-vivo after euthanesia with CO2 according to the guidelines of our institute.

### Antibodies and Reagents

All following conjugated Abs are from BD:APC conjugated anti–mouse CD25, -CD4, -CD45.1, -CD11c, -IL-12p70; Alexa Fluor 700–conjugated anti-CD3, -CD4, and -CD11c; PE-conjugated anti-CD3, -CD19, and -CD49b; FITC-conjugated anti-CD3, -CD19, -CD49b, and isotype control; biotin anti-CD4, -CD8, -DX5, -B220, -CD3, -CD11b, -Ly-6G, and -Ter119; and purified anti-CD16/CD32 (2.4G2). CD11c and streptavidin beads (SA) from Miltenyi Biotec; CFSE, live dead fixable aqua, CL075, and LPS from Invitrogen; ATRA from Sigma-Aldrich; hTGF-β1, anti–mouse TGF-β (1D11), anti-CTLA4, and Ig isotype control from R&D Systems.

### T Cells and DCs

Non-CD4^+^ lymph node and spleen T cells were removed by MACS beads (Miltenyi Biotec) after coating with biotin anti-CD8α, DX5, B220, CD3, CD11b, Ly-6G, and Ter119. Cells were further purified with a FACSAria 2 sorter (BD) to >97%. Spleen CD11c^+^ DCs were partially enriched with anti-CD11c beads (Miltenyi Biotec) and, where indicated, purified with a FACSAria 2 (BD) cell sorter as CD11c^high^CD19^−^CD3^−^DX5^−^ DCs (>95%).

### De Novo In Vitro Induction of T Reg Cells in the Allo-MLR

CD4^+^ T cells from C57BL/6 Foxp3^−^ RFP mice were sorted as CD4^+^CD25^−^RFP^−^ cells. T cells were then co-cultured for 5 d with fresh splenic Balb/c DCs, TGFβ and ATRA as described [11]. Induction of CD4^+^CD25^+^RFP^+^ cells was analyzed by FACS (LSR-II; BD) and FlowJo software (Tree Star) and sorted (FACSAria 2).

### In Vitro IL-12 Induction, Suppression, Consumption and Measurement

DCs from either Balb/c, C57BL/6 or SJL mice were incubated for 24 hrs with either CL075 (1 μg/ml), or LPS (5μg/ml) alone or together with CD4+ T cells. Treg cells were added to the culture for additional 24 hrs in various ratios. IL-12 p70 concentration in the supernatant was measured with ELISA (eBioscience). Intracellular staining for IL-12p70 in DCs or T cells was analyzed by FACS (LSR-II; BD). For consumption assay, DCs, T cells, Treg cells co-incubated or incubated alone for 24 hrs, were cultured with IL-12p70 (eBioscience) and concentration of IL-12p70 remained in the medium was measured by ELISA.

### In Vitro Suppression of T Cell Proliferation and IFNγ Secretion

Effector/activated CD45.1+ CD4+ CD25+ Foxp3^-^ cells were induced from naïve CD4^+^ CD25^-^ in primary MLR co-culture with Balb/c DCs and then sorted, labeled with CFSE and co-cultured with iT reg cells in round-bottom 96-well plates at the indicated ratios plus allo-DCs (ratio of 1 DC to 3 CD45.1^+^ CD4^+^ T cells). After 3 d, proliferation and intracellular IFNγ were assessed by FACS (LSR-II; BD) to determine CFSE diluted, live CD45.1^+^ T cells.

### RTPCR for IL-12Rβ1, β2 and IL-12b

We used the Qiagen kit for RNA isolation from sorted Treg or DCs. SuperScript® Reverse Transcriptase (Invitrogen) was used synthesize cDNA, from which IL-12Rβ1, β2 and IL-12b were amplified with PCR. An agarose gel electrophoresis of the PCR products was performed.

### Single Cell Sort and Analysis

CD4+CD25+Foxp3+(RFP+) cells were sorted from a co-culture of WT iTreg and allo-DCs into 96-well PCR plates (Eppendorf) containing 4μl/well of ice-cold 0.5× PBS supplemented with 10 mM DTT, 8U RNAsin (Promega), and 3U RNAse Inhibitor (5Prime). cDNA was synthesized as described [36], and nested PCR for IL-12Rβ2 and Foxp3 cDNA was performed.

The following primers were used to amplify gene-specific cDNA fragments from the single cells: IL-12Rβ2 outer-down, 5’-CCC TTG GTA TGA CCT TGT TTG TCT GCA AGC-3’; IL-12Rβ2 outer-up, 5’-GAT CCA GGC GAT TGC AAT TCT GAC GAT TGT CA-3’; IL-12Rβ2 inner-down, 5’-AAA AGA AGC CAC CAG TCC CAG TAT G-3’; IL-12Rβ2 inner-up, 5’-CTG ACA GGT CAG ATT GTT TGG TCC A-3’; Foxp3 outer-down, 5’-TTC CAA GGT CGG GAC CTG CGA AGT GGG G-3’; Foxp3 outer-up, 5’-GCA GAA GGT GGT GGG AGG CTG ATC ATG GCT-3’; Foxp3 inner-down, 5’-ACA CCT CTT CTT CCT TGA ACC CCC TG-3’; Foxp3 inner-up, 5’-TCC AGT GGA CGC ACT TGG AGC ACA-3’.

**Statistical** data were analyzed using Student’s *t* test.

## Supporting Information

S1 Fig DCs generate *de-novo* CD4+ CD25+ Foxp3+ (RFP+) from a polyclonal T cell repertoire in the mixed leukocyte reaction (MLR). Sorted C57Bl/6 CD4+ CD25- Foxp3-(RFP-) spleen cells were labeled with CFSE and cultured 5 d with Balb/c splenic CD11c+ DCs without (left panel) or with (right panel) TGFb (20 ng/ml) and ATRA (10nM). Shown is the frequency of induced CD4+ CD25+ Foxp3+ (RFP+) iTreg by FACS analysis on d5. One representative experiment of three is shown. Adapted from “Dendritic cells induce antigen-specific regulatory T cells that prevent graft versus host disease and persist in mice”. By Sela U et al, *J*. *Exp*. *Med*. 208:2489–2496.
(TIF)

S2 Fig DC induced Treg have mild effect on TNFα secreted from DC. Balb/c DCs were incubated (24hr) either alone or with various ratio of C57Bl/6 iTreg that were induced with Balb/c DCs (iTreg anti Balb). Then the culture was added with CL075 (1 mg/ml) and 24 hr later level of TNFα secreted to the medium was measured with ELISA. One representative experiment of three is shown. Error bars denote mean ± SD. *, P < 0.05.
(TIF)

S3 Fig The main source for IL-12 in the MLR culture are DCs. Balb/c DCs or C57Bl/6 CD4+ T cells were cultured alone or together (MLR) and incubated alone or co-incubated with CL075 (24 hr). Level of IL-12p70 secreted to the medium was measured by ELISA (left panel) or the culture was stained, and analyzed by FACS for IL-12 positive T (CD3+CD4+) or dendritic (CD11c+) cells. One representative experiment of three is shown. Error bars denote mean ± SD. * = comparing CL075 induced to non-induced DCs, P < 0.05, ** = comparing CL075 induced to non-induced co-culture of DCs and CD4+ T cells. P < 0.05.
(TIF)

S4 Fig DC induced Treg have mild effect on TNFα and IL-6 secreted from MLR of CD4+ T cells and DCs. MLR of Balb/c DCs and C57Bl/6 CD4+ T cells were incubated (24hr) either alone or with various ratio of C57Bl/6 iTreg that were induced with Balb/c DCs (iTreg anti Balb). Then, CL075 (left panel) or LPS (5 mg/ml, right panel) were added to the culture, and 24 hr later level of TNFα (left panel) or IL-6 (right panel) secreted to the medium was measured by ELISA. One representative experiment of three is shown. Error bars denote mean ± SD. *, P < 0.05.
(TIF)

S5 Fig DC induced Treg specifically down-regulate IL-12 secreted from allo-DCs. Balb/c, C57Bl/6, or F1 (Balb/c X B6) DCs were incubated (24hr) either alone, with C57Bl/6 CD4+ T cells or with C57Bl/6 CD4+ T cells together with various ratio of C57Bl/6 iTreg that were induced with Balb/c DCs (iTreg anti Balb). Then CL075 was added to the culture, and 24 hr later level of IL-12 secreted to the medium was compared by ELISA. One representative experiment of three is shown. Error bars denote mean ± SD. *, P < 0.05 (comparing to DC+T+CL0 without iTreg).
(TIF)

S6 Fig iTreg down-regulate CL075 induced IL-12 secreted from DCs in MHC class-II dependent manner. Balb/c DCs were incubated (24hr) either alone, with C57Bl/6 CD4+ T cells or with C57Bl/6 CD4+ T cells and C57Bl/6 iTreg at 1 to 1 ratio. Either anti-MHC-class II (upper panel), anti-CTLA-4 (left lower panel), or anti-TGFβ (right lower panel) mAbs (10mg/ml) or their isotype control (IC) were added or not to the culture containing the iTreg cells. Then the culture was added with CL075 and 24 hr later level of IL-12 secreted to the medium was compared with ELISA. One representative experiment of three is shown. Error bars denote mean ± SD. *, P < 0.05 (compared to IC).
(TIF)

S7 Fig The down-regulation of IL-12 secreted from DCs by iTreg is not due to marked DC death nor inhibition of its production. (A) Co-culture of DCs and CD4+ T cells were incubated either alone or co-incubated with iTreg cells for 36 hrs after which the culture was stained for viability (aqua) and compared by FACS for live CD11c+ cells. (B) As in A, but the cultured DCs (CD11c+) were sorted, and RNA was isolated. A gel of PCR products of IL-12p40 cDNA is shown. (C) As in A, but DCs are from IL-12 p40 reporter C57Bl/6 mice, and the culture was stimulated with CL075 and analyzed by FACS for IL-12 expressing DCs (CD11c+). One representative experiment of three is shown.
(TIF)

S8 Fig DC induced Treg cells have minor effect on consumption of TNFα from the medium. TNFα (1000pg/ml) in culture medium was incubated alone or with either DCs, CD4+ T cells, or iTreg alone or with a co-culture of DCs and either CD4+ or anti Balb iTreg. After 48hrs level of TNFα that remained in the medium was measured by ELISA. One representative experiment of three is shown.
(TIF)

S9 Fig IL-12 enhances suppression of T cell proliferation by iTreg. (A) MLR culture of Balb/c DCs and sorted CFSE labeled effector CD4^+^CD25^+^Foxp3^-^cells (following naïve CD4^+^CD25^-^ pre-activation with Balb/c DCs for 3 days) was incubated without or with IL-12 (1000 pg/ml) or various concentration of WT iTreg cells for 3 days and analyzed by FACS. Absolute number (upper panel) of effector proliferating cells and their percent (lower panel) out of the MLR (considered as 100%) are shown. (B) As in A, but the MLR culture was added by either WT or IL-12Rβ2 KO iTreg cells. Absolute number (middle panel), percent of effector proliferating cells (left panel) and FACS histogram of CFSE dilution (right panel), are shown.One representative experiment of three is shown. Error bars denote mean ± SD. *, P < 0.05 (compared to WT iTreg).
(TIF)

S10 Fig IL-12Rβ2 mediates IL-12 induced IFNγ secretion from iTreg. Balb/c DCs were cultured with either WT or IL-12Rβ2 KO sorted anti Balb/c iTreg. The culture was either stimulated (left lower panels) or not stimulated (left upper panels) with IL-12, and 24 hrs later stained and analyzed (left panel) for IFNγ expressing Treg cells (CD4+CD25+Foxp3+(RFP+) by FACS (absolute number in right upper panel), and for IFNγ secreted to the medium by ELISA (right lower panel). One representative experiment of three is shown.
(TIF)
