# Supplementary figures and images for: Dendritic Cells Induce a Subpopulation of IL-12Rβ2-Expressing Treg that Specifically Consumes IL-12 to Control Th1 Responses
Source: PLoS One. 2016 Jan 8;11(1):e0146412. doi: 10.1371/journal.pone.0146412 (PMC4706322; doi:10.1371/journal.pone.0146412)

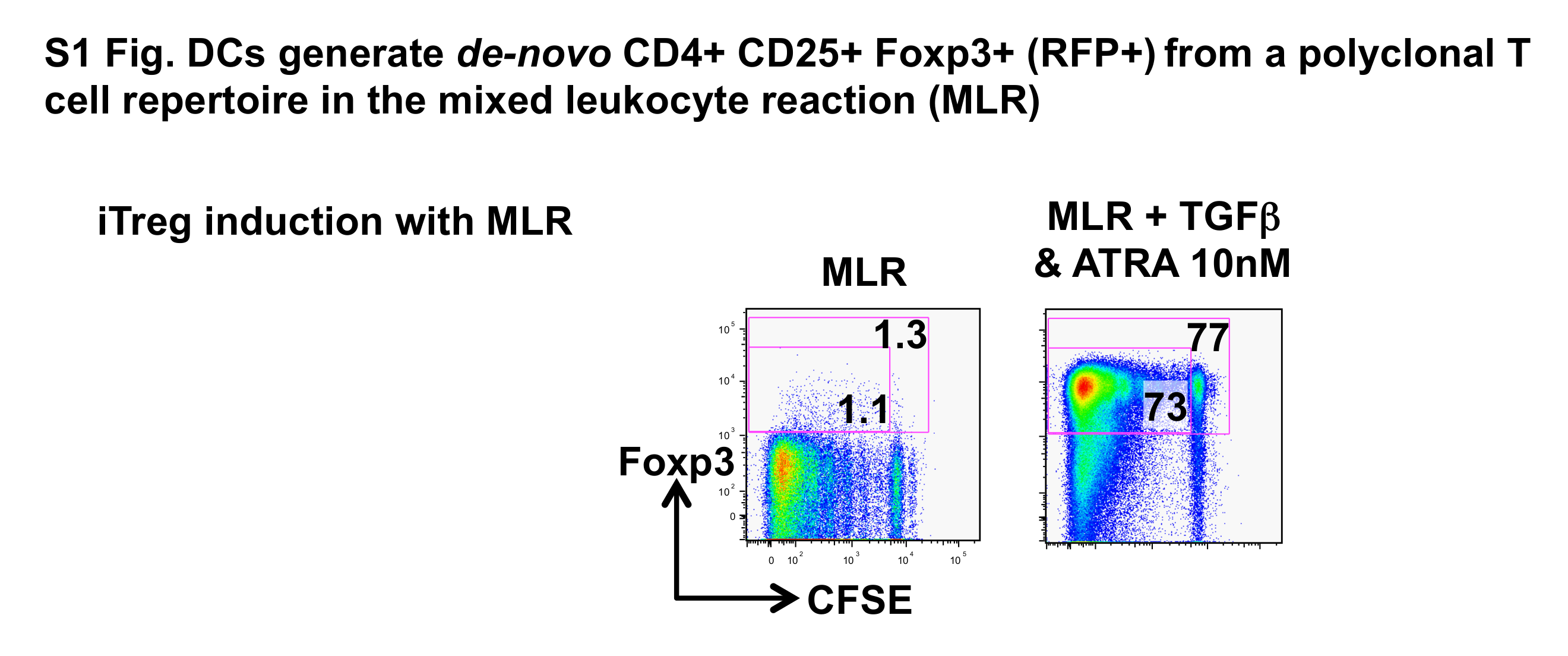

Supplement: S1 Fig — Sorted C57Bl/6 CD4+ CD25- Foxp3-(RFP-) spleen cells were labeled with CFSE and cultured 5 d with Balb/c splenic CD11c+ DCs without (left panel) or with (right panel) TGFb (20 ng/ml) and ATRA (10nM). Shown is the frequency of induced CD4+ CD25+ Foxp3+ (RFP+) iTreg by FACS analysis on d5. One representative experiment of three is shown. Adapted from “Dendritic cells induce antigen-specific regulatory T cells that prevent graft versus host disease and persist in mice”. By Sela U et al, J. Exp. Med. 208:2489–2496. (TIF) [file pone.0146412.s001.tif]

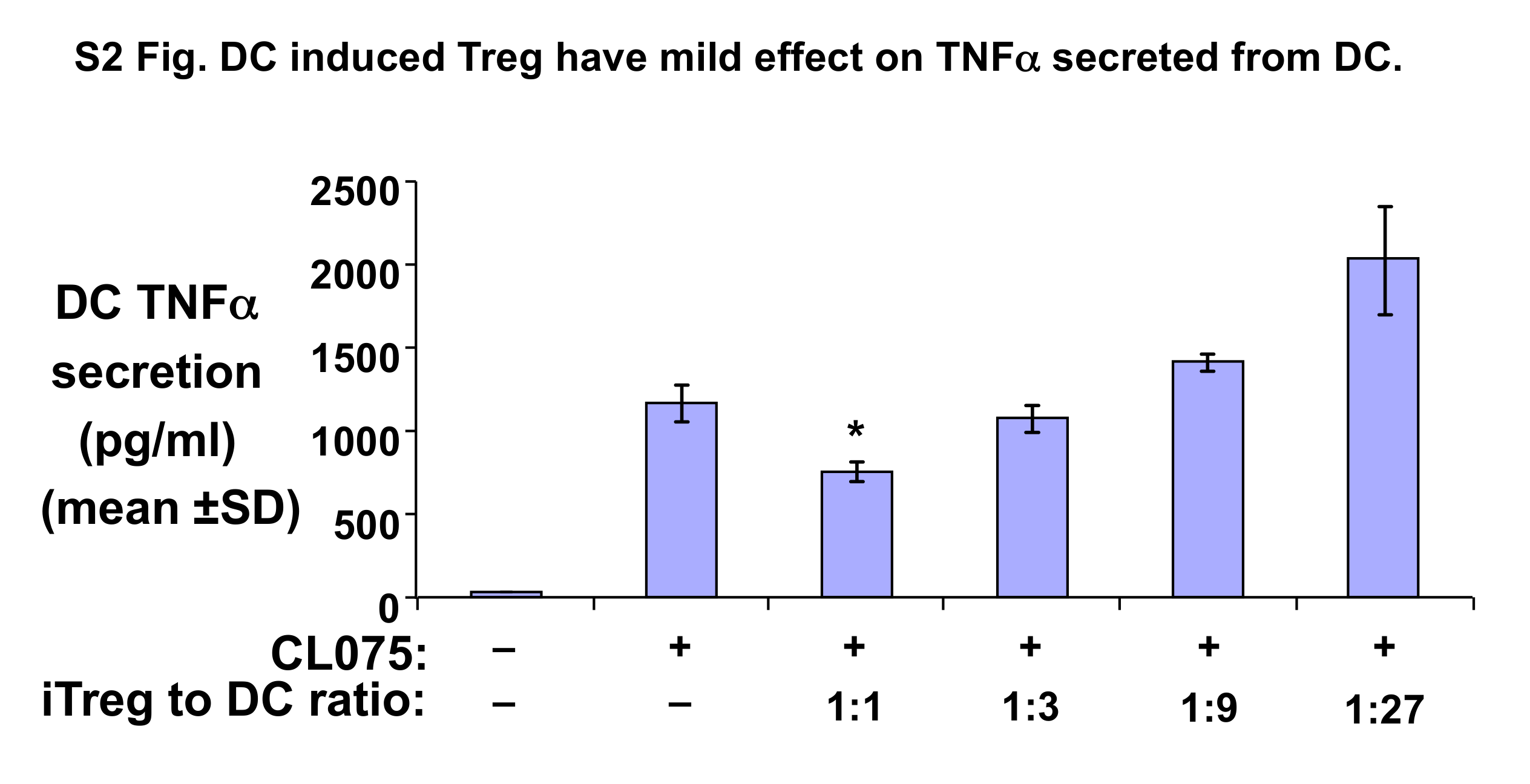

Supplement: S2 Fig — Balb/c DCs were incubated (24hr) either alone or with various ratio of C57Bl/6 iTreg that were induced with Balb/c DCs (iTreg anti Balb). Then the culture was added with CL075 (1 mg/ml) and 24 hr later level of TNFα secreted to the medium was measured with ELISA. One representative experiment of three is shown. Error bars denote mean ± SD. *, P < 0.05. (TIF) [file pone.0146412.s002.tif]

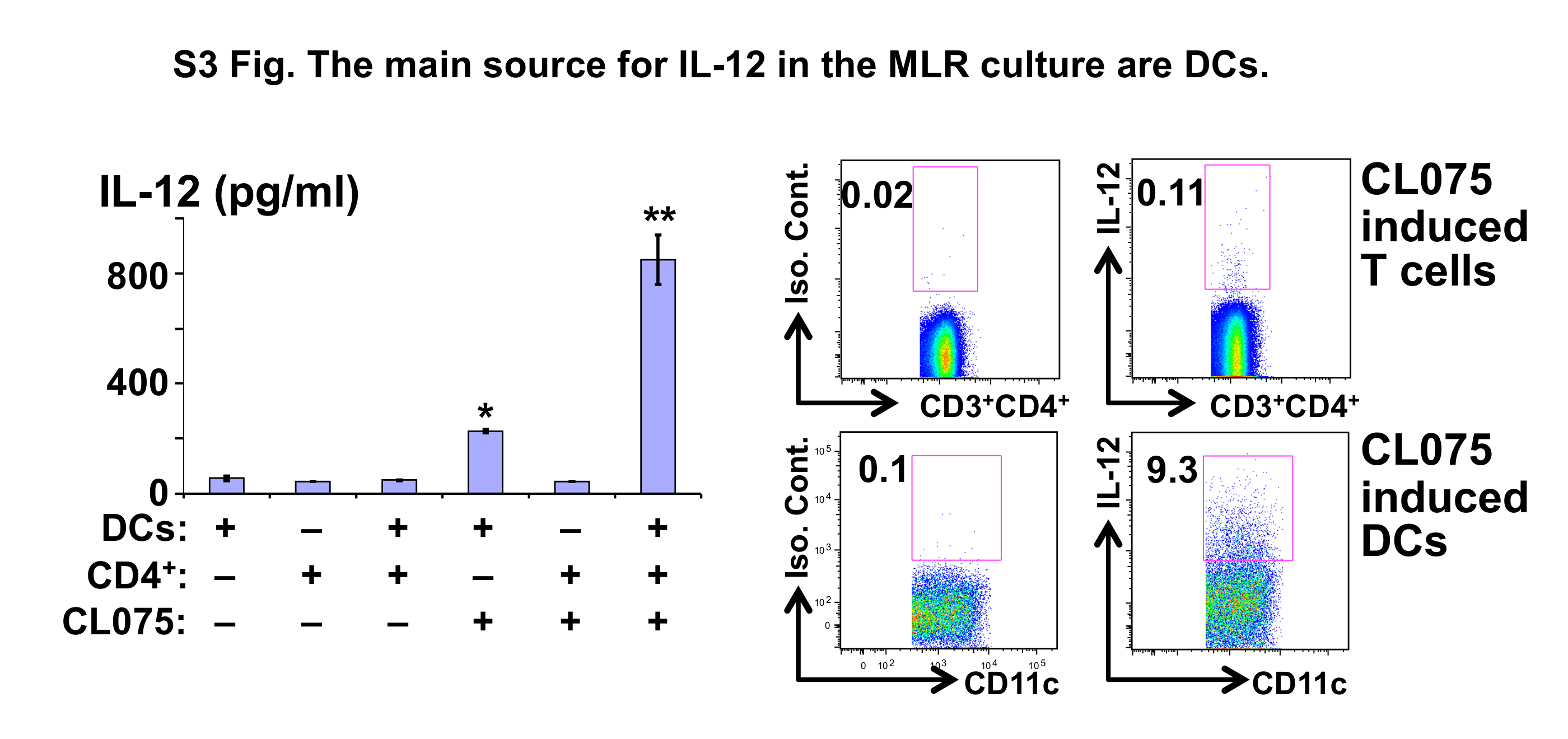

Supplement: S3 Fig — Balb/c DCs or C57Bl/6 CD4+ T cells were cultured alone or together (MLR) and incubated alone or co-incubated with CL075 (24 hr). Level of IL-12p70 secreted to the medium was measured by ELISA (left panel) or the culture was stained, and analyzed by FACS for IL-12 positive T (CD3+CD4+) or dendritic (CD11c+) cells. One representative experiment of three is shown. Error bars denote mean ± SD. * = comparing CL075 induced to non-induced DCs, P < 0.05, ** = comparing CL075 induced to non-induced co-culture of DCs and CD4+ T cells. P < 0.05. (TIF) [file pone.0146412.s003.tif]

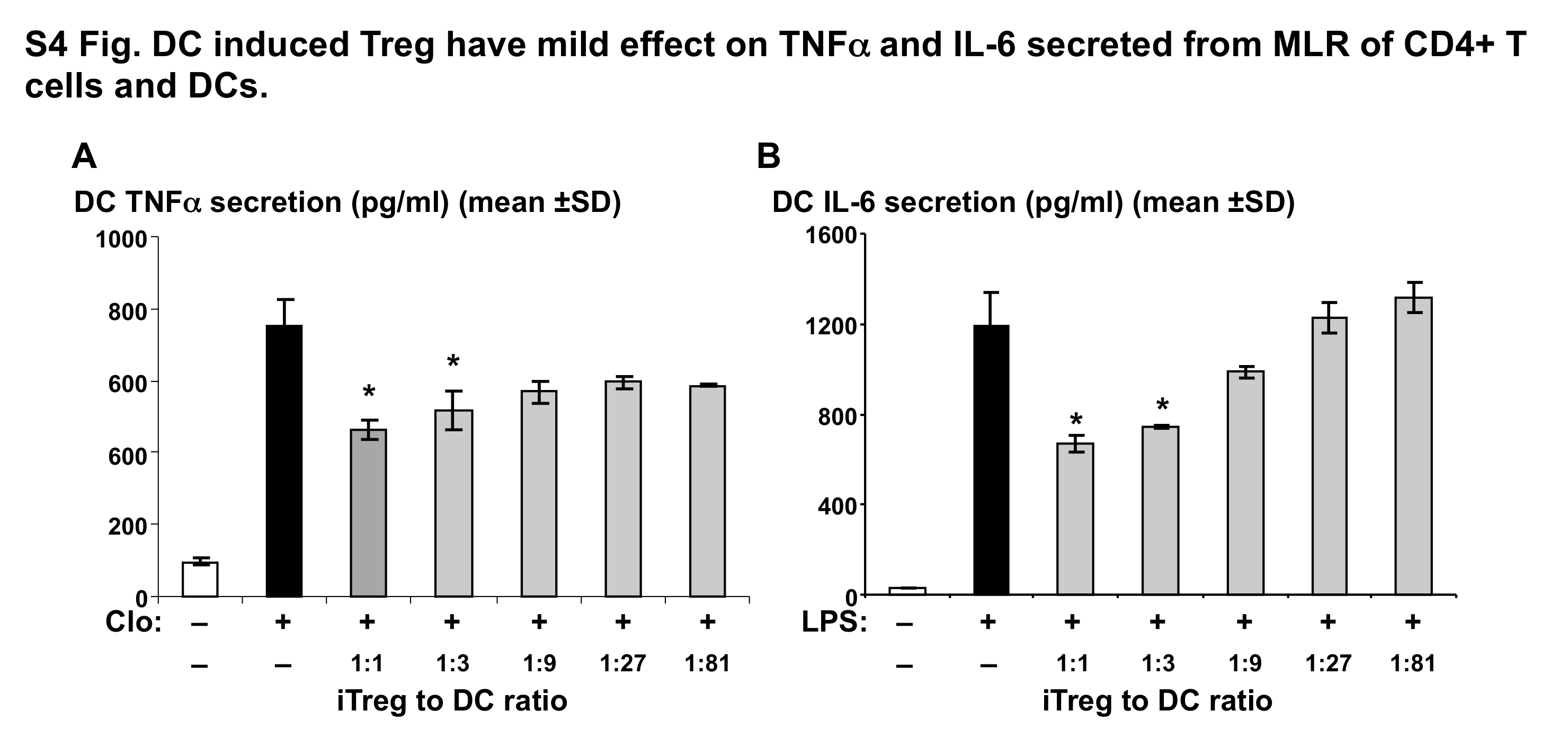

Supplement: S4 Fig — MLR of Balb/c DCs and C57Bl/6 CD4+ T cells were incubated (24hr) either alone or with various ratio of C57Bl/6 iTreg that were induced with Balb/c DCs (iTreg anti Balb). Then, CL075 (left panel) or LPS (5 mg/ml, right panel) were added to the culture, and 24 hr later level of TNFα (left panel) or IL-6 (right panel) secreted to the medium was measured by ELISA. One representative experiment of three is shown. Error bars denote mean ± SD. *, P < 0.05. (TIF) [file pone.0146412.s004.tif]

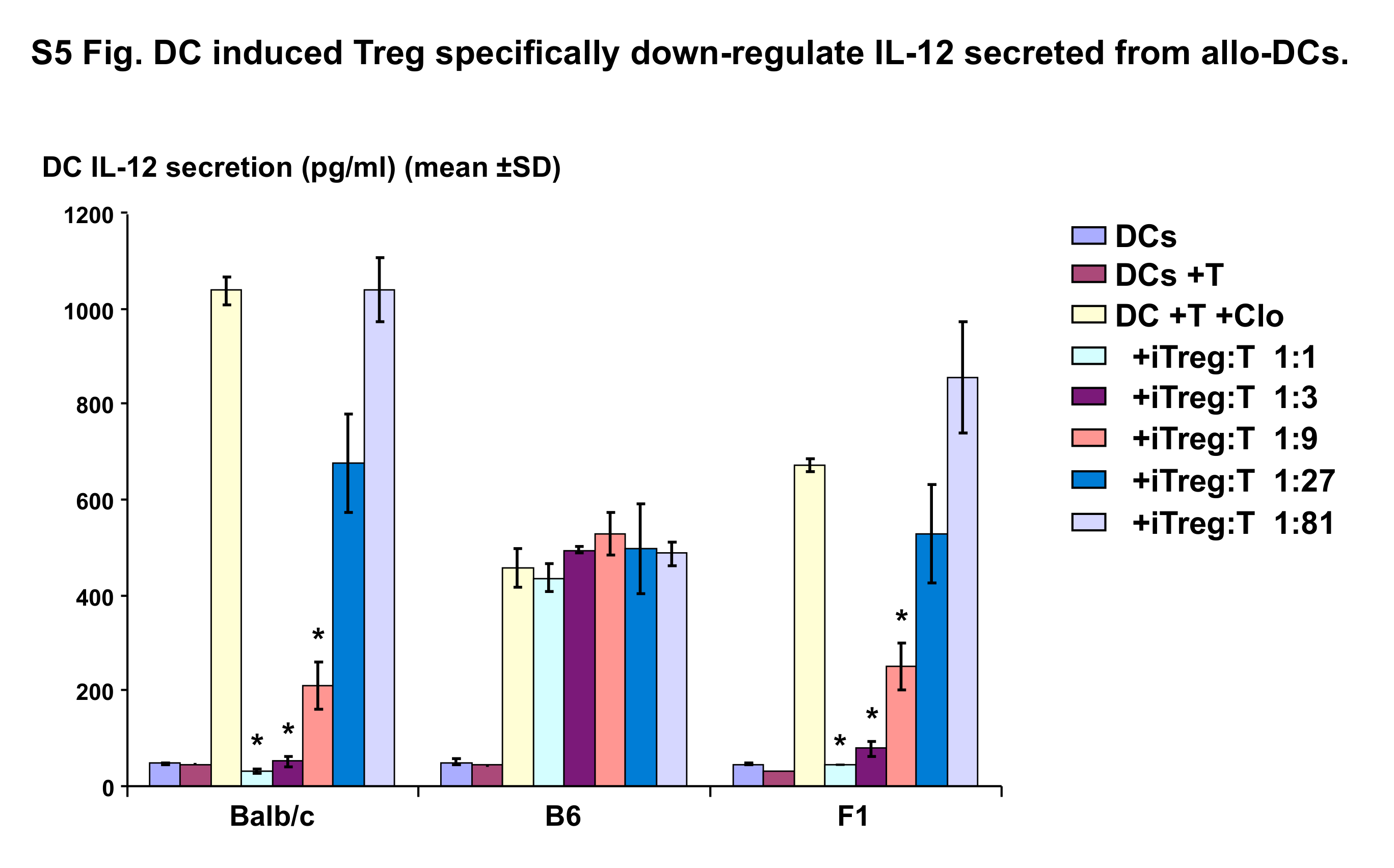

Supplement: S5 Fig — Balb/c, C57Bl/6, or F1 (Balb/c X B6) DCs were incubated (24hr) either alone, with C57Bl/6 CD4+ T cells or with C57Bl/6 CD4+ T cells together with various ratio of C57Bl/6 iTreg that were induced with Balb/c DCs (iTreg anti Balb). Then CL075 was added to the culture, and 24 hr later level of IL-12 secreted to the medium was compared by ELISA. One representative experiment of three is shown. Error bars denote mean ± SD. *, P < 0.05 (comparing to DC+T+CL0 without iTreg). (TIF) [file pone.0146412.s005.tif]

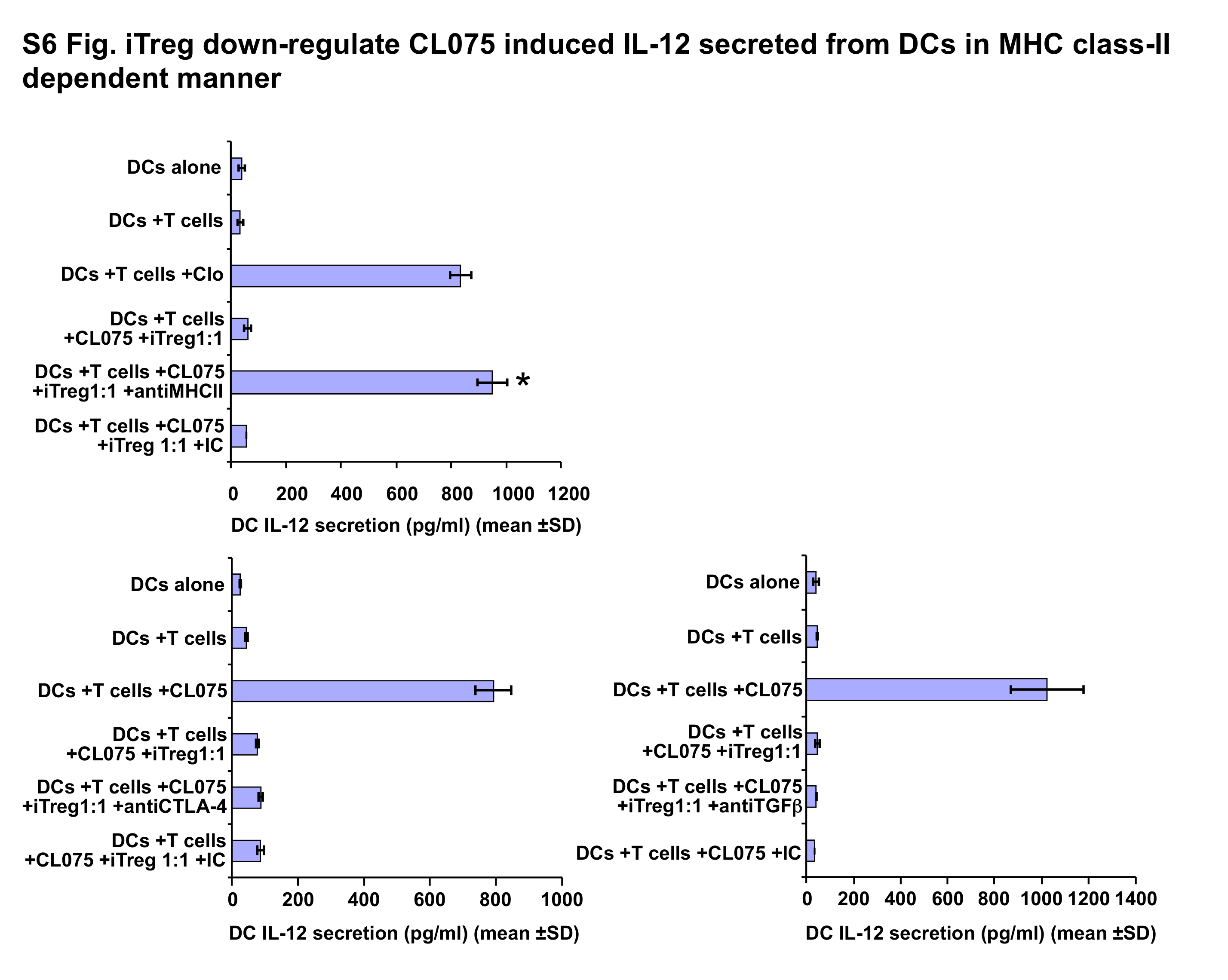

Supplement: S6 Fig — Balb/c DCs were incubated (24hr) either alone, with C57Bl/6 CD4+ T cells or with C57Bl/6 CD4+ T cells and C57Bl/6 iTreg at 1 to 1 ratio. Either anti-MHC-class II (upper panel), anti-CTLA-4 (left lower panel), or anti-TGFβ (right lower panel) mAbs (10mg/ml) or their isotype control (IC) were added or not to the culture containing the iTreg cells. Then the culture was added with CL075 and 24 hr later level of IL-12 secreted to the medium was compared with ELISA. One representative experiment of three is shown. Error bars denote mean ± SD. *, P < 0.05 (compared to IC). (TIF) [file pone.0146412.s006.tif]

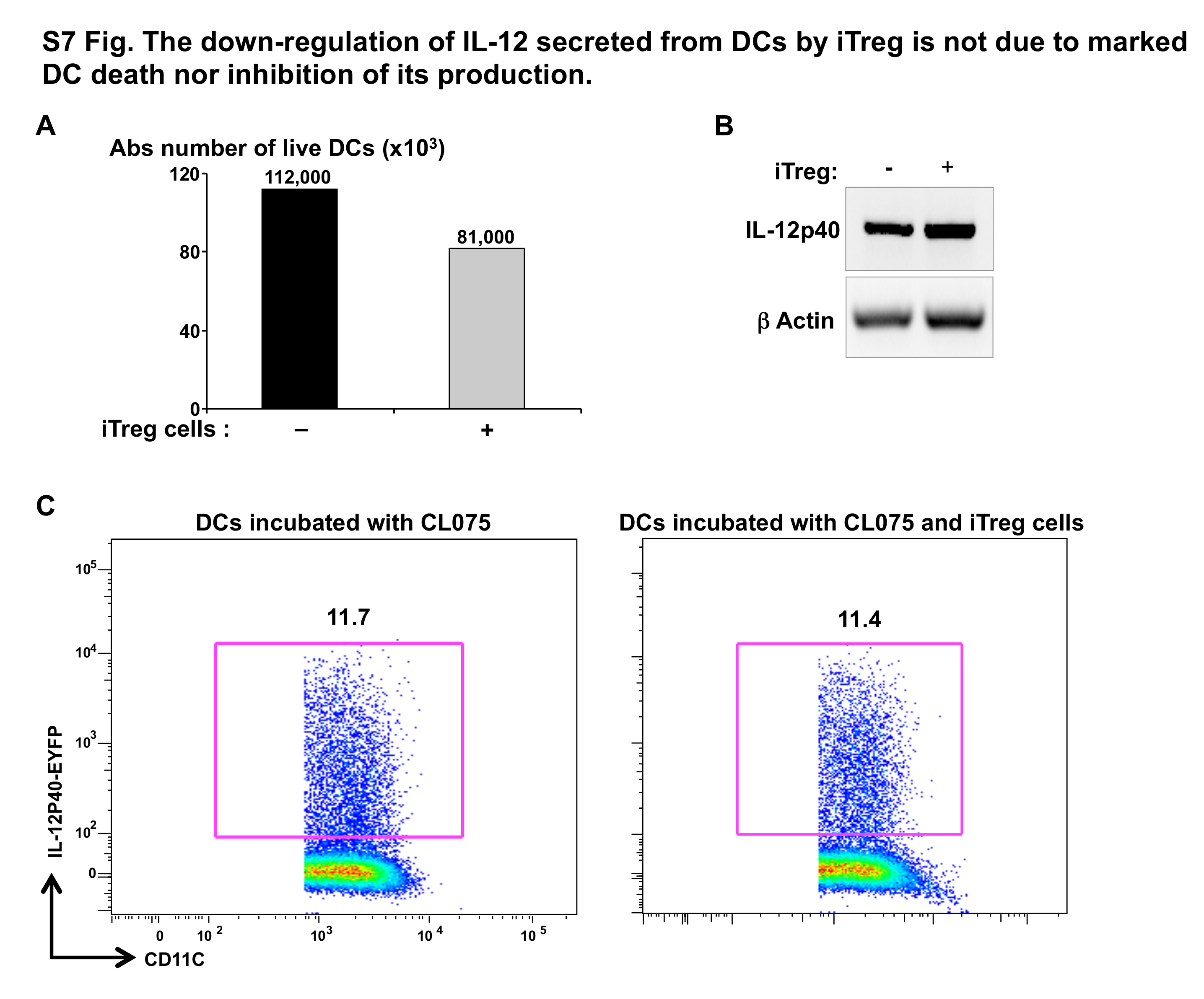

Supplement: S7 Fig — (A) Co-culture of DCs and CD4+ T cells were incubated either alone or co-incubated with iTreg cells for 36 hrs after which the culture was stained for viability (aqua) and compared by FACS for live CD11c+ cells. (B) As in A, but the cultured DCs (CD11c+) were sorted, and RNA was isolated. A gel of PCR products of IL-12p40 cDNA is shown. (C) As in A, but DCs are from IL-12 p40 reporter C57Bl/6 mice, and the culture was stimulated with CL075 and analyzed by FACS for IL-12 expressing DCs (CD11c+). One representative experiment of three is shown. (TIF) [file pone.0146412.s007.tif]

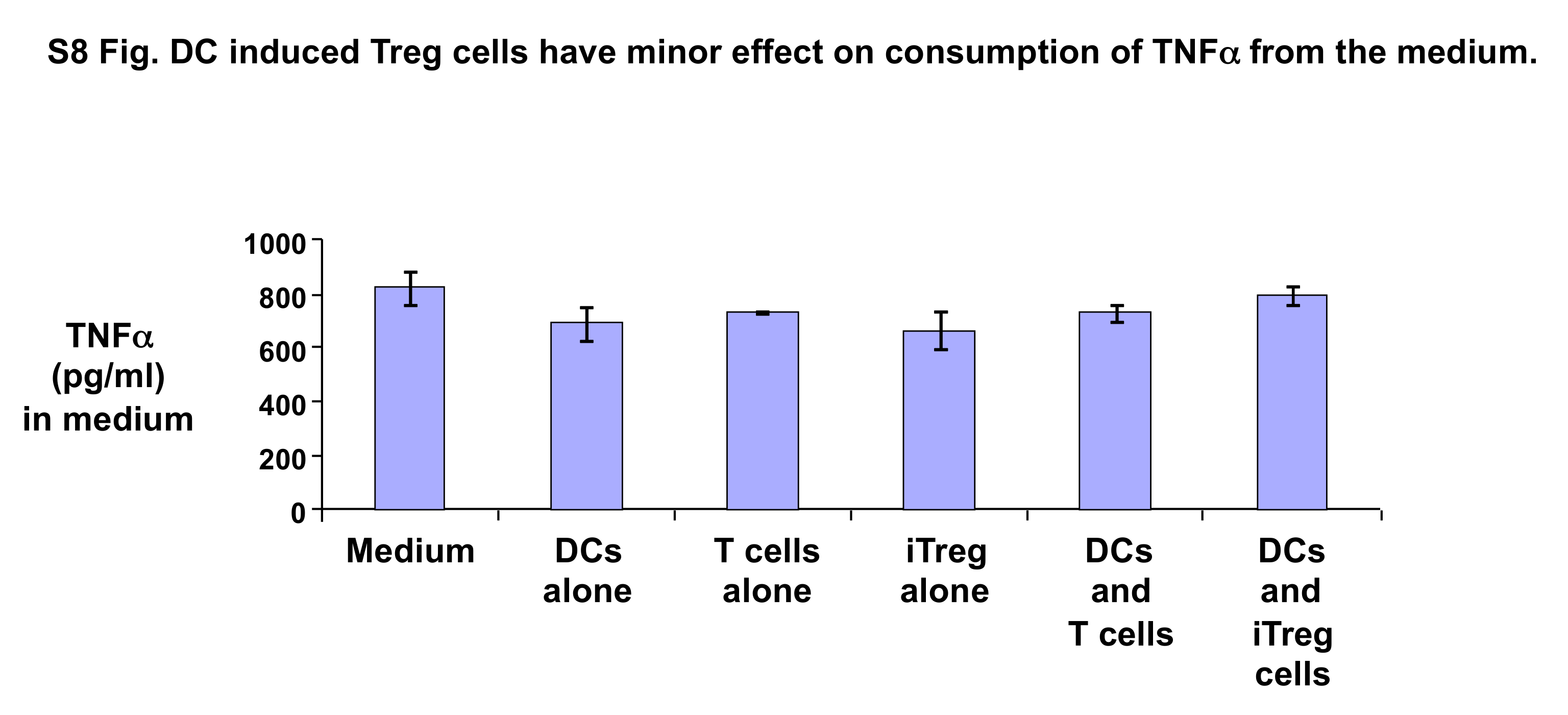

Supplement: S8 Fig — TNFα (1000pg/ml) in culture medium was incubated alone or with either DCs, CD4+ T cells, or iTreg alone or with a co-culture of DCs and either CD4+ or anti Balb iTreg. After 48hrs level of TNFα that remained in the medium was measured by ELISA. One representative experiment of three is shown. (TIF) [file pone.0146412.s008.tif]

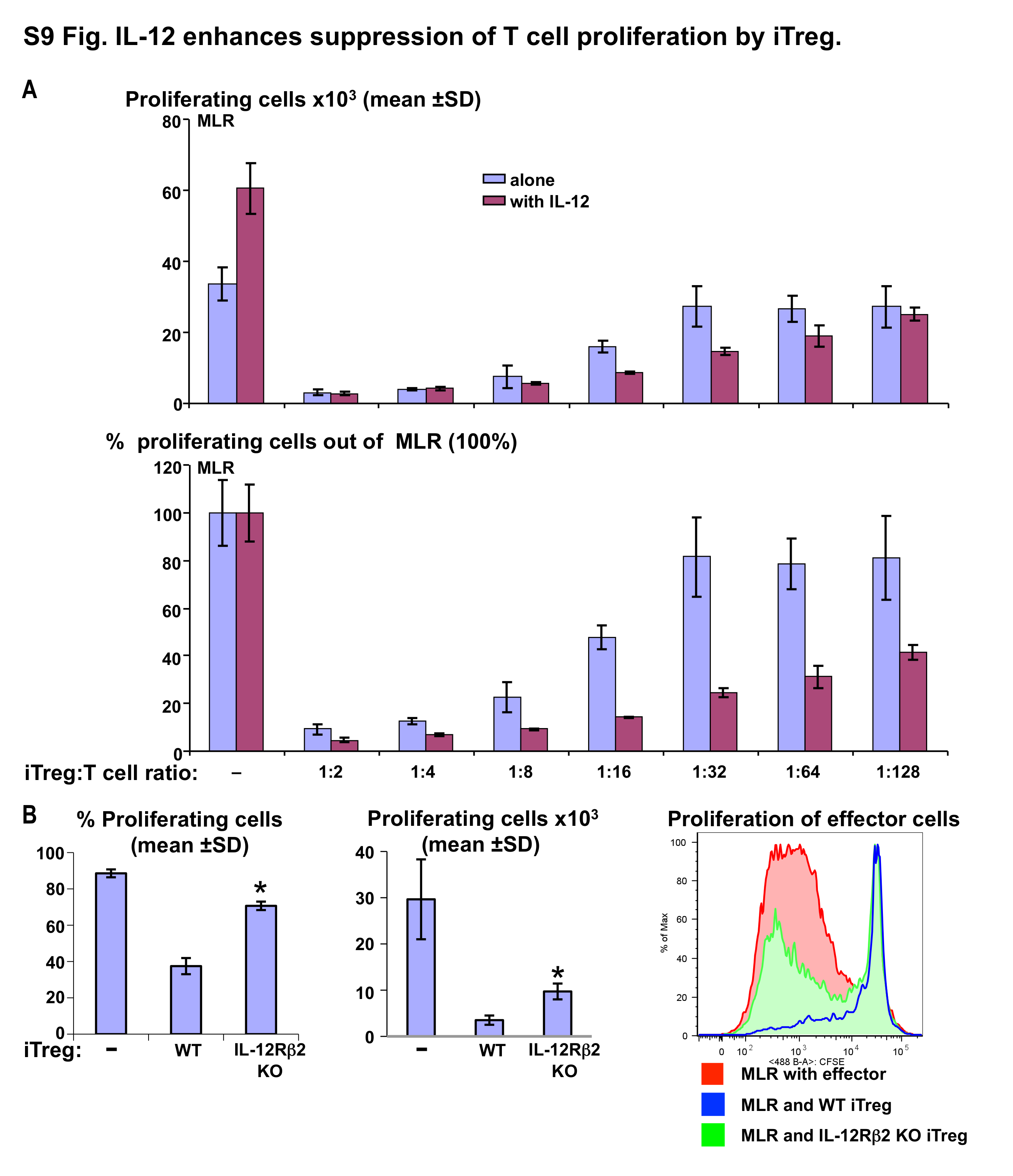

Supplement: S9 Fig — (A) MLR culture of Balb/c DCs and sorted CFSE labeled effector CD4+CD25+Foxp3-cells (following naïve CD4+CD25- pre-activation with Balb/c DCs for 3 days) was incubated without or with IL-12 (1000 pg/ml) or various concentration of WT iTreg cells for 3 days and analyzed by FACS. Absolute number (upper panel) of effector proliferating cells and their percent (lower panel) out of the MLR (considered as 100%) are shown. (B) As in A, but the MLR culture was added by either WT or IL-12Rβ2 KO iTreg cells. Absolute number (middle panel), percent of effector proliferating cells (left panel) and FACS histogram of CFSE dilution (right panel), are shown.One representative experiment of three is shown. Error bars denote mean ± SD. *, P < 0.05 (compared to WT iTreg). (TIF) [file pone.0146412.s009.tif]

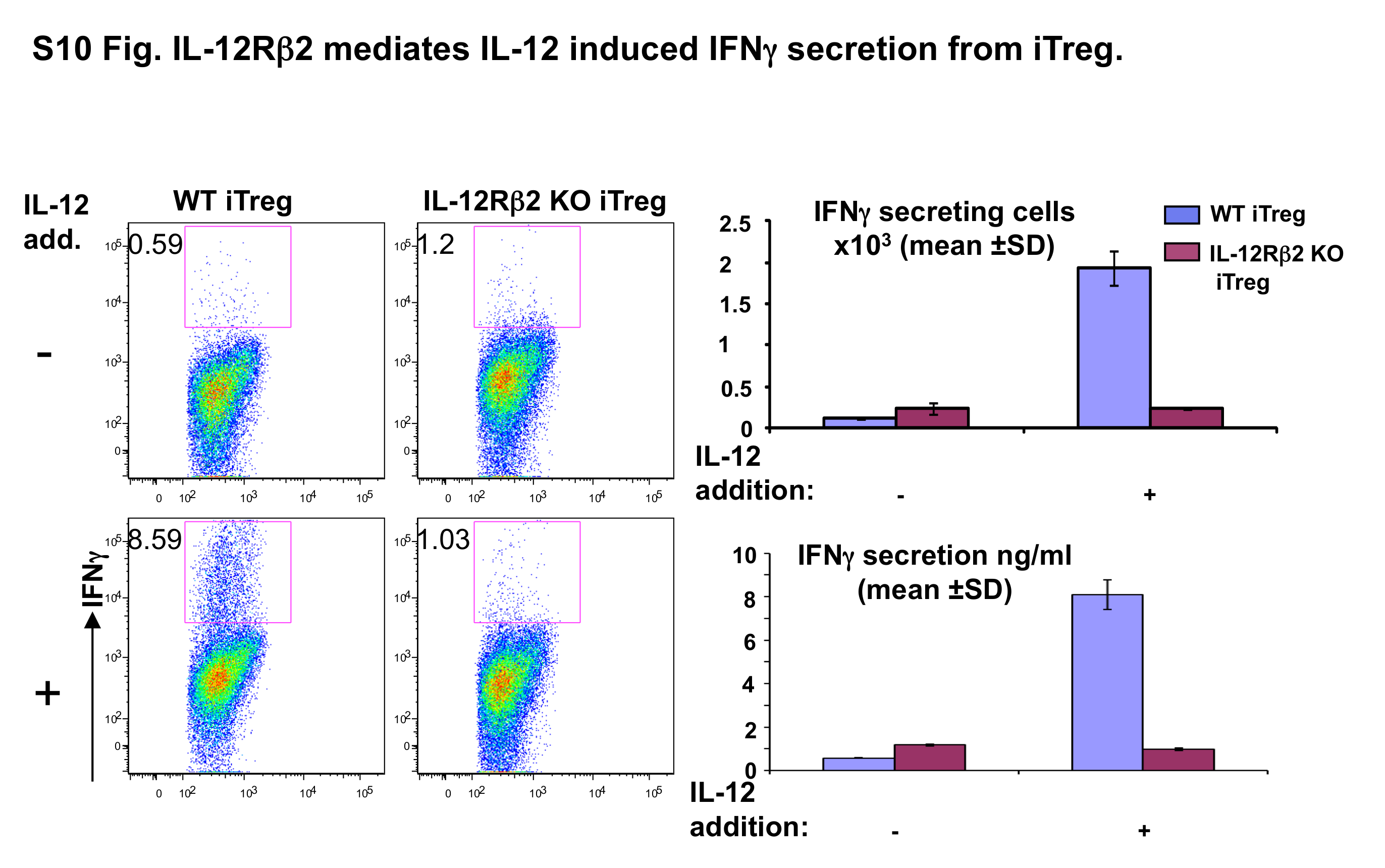

Supplement: S10 Fig — Balb/c DCs were cultured with either WT or IL-12Rβ2 KO sorted anti Balb/c iTreg. The culture was either stimulated (left lower panels) or not stimulated (left upper panels) with IL-12, and 24 hrs later stained and analyzed (left panel) for IFNγ expressing Treg cells (CD4+CD25+Foxp3+(RFP+) by FACS (absolute number in right upper panel), and for IFNγ secreted to the medium by ELISA (right lower panel). One representative experiment of three is shown. (TIF) [file pone.0146412.s010.tif]
